# Numerical Reconstruction of Cyclist Impact Accidents: Can Helmets Protect the Head-Neck of Cyclists?

**DOI:** 10.3390/biomimetics8060456

**Published:** 2023-09-27

**Authors:** Fang Wang, Ke Peng, Tiefang Zou, Qiqi Li, Fan Li, Xinghua Wang, Jiapeng Wang, Zhou Zhou

**Affiliations:** 1 School of Automotive and Mechanical Engineering, Changsha University of Science and Technology, Changsha 410114, China; wangfang83715@163.com (F.W.); ikun_502@163.com (K.P.); tiefang@163.com (T.Z.); hdliqiqi@163.com (Q.L.); 15719951195@163.com (J.W.); 2 Hunan Province Key Laboratory of Safety Design and Reliability Technology for Engineering Vehicle, Changsha University of Science and Technology, Changsha 410114, China; 3 State Key Laboratory of Advanced Design and Manufacturing for Vehicle Body, Hunan University, Changsha 410082, China; lifandudu@163.com; 4 Division of Neuronic Engineering, KTH Royal Institute of Technology, 14152 Stockholm, Sweden; zhouz@kth.se

**Keywords:** numerical simulation, cyclist impact accident, helmet, head/brain injury, neck injury

## Abstract

Cyclists are vulnerable road users and often suffer head-neck injuries in car–cyclist accidents. Wearing a helmet is currently the most prevalent protection method against such injuries. Today, there is an ongoing debate about the ability of helmets to protect the cyclists’ head-neck from injury. In the current study, we numerically reconstructed five real-world car–cyclist impact accidents, incorporating previously developed finite element models of four cyclist helmets to evaluate their protective performances. We made comparative head-neck injury predictions for unhelmeted and helmeted cyclists. The results show that helmets could clearly lower the risk of severe (AIS 4+) brain injury and skull fracture, as assessed by the predicted head injury criterion (HIC), while a relatively limited decrease in AIS 4+ brain injury risk can be achieved in terms of the analysis of CSDM_0.25_. Assessment using the maximum principal strain (MPS_0.98_) and head impact power (HIP) criteria suggests that helmets could lower the risk of diffuse axonal injury and subdural hematoma of the cyclist. The helmet efficacy in neck protection depends on the impact scenario. Therefore, wearing a helmet does not seem to cause a significant neck injury risk level increase to the cyclist. Our work presents important insights into the helmet’s efficacy in protecting the head-neck of cyclists and motivates further optimization of protective equipment.

## 1. Introduction

China ranks first in the world with more than 400 million bicycles [1], yet cyclists are particularly vulnerable in road traffic accidents [1]. In this country, cyclists account for the highest ratio of fatalities and injuries among all types of road users [2]. Head injury is the most frequently observed trauma type in cyclist-related road accidents with brain injury being the predominant cause of cyclist mortality [3,4,5,6,7].

Many cyclists use a helmet while riding. At present, approaches for the evaluation of helmet protection performance mainly include the physical drop test [8,9,10,11,12,13] and computational simulation via finite element (FE) models [12,13,14,15,16]. Initially, the helmet drop test method was used, in which a helmeted headform was dropped onto a flat/declined surface with kinematic peaks (e.g., maximum linear acceleration at the headform’s center of gravity) less than certain thresholds [12]. Later, the introduction of the computational modeling method made it much more feasible/efficient to comprehensively investigate the ability of helmets in terms of reducing the head/brain injury risk in diverse loadings. Bourdet et al. [12] derived the initial conditions of cyclists’ heads from the reconstruction of several car–cyclist impact cases and calculated the risk of head/brain injury by using the FE head-only model. Xiao et al. [16] analyzed the influence of impact conditions such as angle and velocity on brain injury parameters with both multibody (MB) and finite element (FE) computational models. Similar to the research by Bourdet et al. [12], they simulated a series of car-to-cyclist impacts to obtain diverse boundary conditions (impact position, orientation and velocity) of the head when head–vehicle impact occurs, and input these conditions to a head FE model to obtain the brain tissue level responses. The results showed a clear reduction in skull stress for the helmeted cyclists. As it may be a compromised strategy to circumvent the challenges of performing full-scale FE human body simulations (e.g., high computation cost, low efficiency of adjusting the cyclist posture), both studies used the head-only model to predict the head/brain injury responses in helmeted impacts. Despite interesting initial results, such an approach suffers critical limitations mainly from two aspects [17,18,19,20,21]: Firstly, in car–cyclist impact accidents, the head of the cyclist typically endures two impact stages, in which the first impact occurs with the car exterior (e.g., the windscreen) and the secondary one with the ground. The head-only simulation is not able to replicate the second impact stage with the ground [17,18,19,20,22]. This is partially evidenced by the fact that existing research work has largely focused on the first impact alone [23,24,25]. Secondly, for the head-only simulation, the calculated kinematics-based head injury responses (HIC_15_, linear and angular acceleration of the head center of gravity COG) are notably lower than those obtained using the full-scale human body model, which suggests that ignoring other body segments may be inadequate [20,26].

In comparison to the growing body of helmet-related studies that focused on the head injuries of cyclists, the influence of wearing a helmet on the cyclist’s neck was largely ignored. This is evidenced by the fact that, in most medical records of traffic accidents, the neck soft tissue injuries are often not documented [27]. For example, Kasantikul et al. [28] conducted a dissection of the neck of 73 motorcycle riders and found that neck soft tissue injuries were concealed in some cases regardless of the use of the helmet, and were not included in the medical records. Among the handful of studies about the neck region, most of them are based on case-control studies and anatomical investigation [27,29,30]. Kim et al. and Emamgholipour et al. found that neck injuries are common in VRU-related traffic accidents, but that most neck injuries are not serious [31,32]. Amoros et al. [29] confirmed the ability of helmets to prevent head and facial injuries using a case-control study. However, the helmet’s efficacy in reducing neck injury risk was inconclusive. In a meta-analysis study, Hoye [30] reported the protective performance of helmets against serious head injury due to bicycle impact, while helmets had no statistically significant effect on preventing cervical spine injury. These inconsistencies highlight the need for research to evaluate the helmet’s efficacy in neck protection.

This paper investigates the ability of cycle helmets to prevent head/brain and neck injuries due to car impact accidents. The research is conducted via computational reconstruction of five actual car–cyclist crash accidents, and comparative analysis of the head/brain and neck injury responses/risks between helmeted and unhelmeted cyclists.

## 2. Materials and Methods

### 2.1. Accident Information

Five car–cyclist impact cases used here are from the In-Depth Investigation of Vehicle Accident in Changsha (IVAC) database [1], which was founded by Hunan University, Changsha, China, aiming to gather on-site data on real-world traffic accident cases in the city of Changsha, China. The principles for selecting the accident include: the case can be used for research purposes; the cyclist was not wearing a helmet when the accident occurred; the cyclist is not younger than 18, not shorter than 1.5 m, and sustained head impact with the car windshield; the information on the accident is appropriate for numerical reconstruction. The information about the five selected accidents is shown in Table 1.

### 2.2. Accident Numerical Reconstruction

The selected accidents were reconstructed using the vehicle/bicycle and subject-specific cyclist MB models. The MB models were performed using a MADYMO V7.7 MB software package from TASS (Helmond, The Netherlands: http://www.tassinternational.com/madymo, accessed on 24 September 2023), which is widely utilized in the field of vehicle safety and injury biomechanics. We followed our previous research for the specific accident reconstruction process [20,33,34]. For the reconstruction, information about the initial velocity of the vehicle, relative position of the cyclist and vehicle before the accident, and the cyclist’s posture, were firstly obtained from the database; the parameters describing the prior-to-accident situation were then calibrated so that the position of the cyclist and vehicle as well as the impact location of the cyclist and vehicle calculated from the reconstruction agree with those observed in the accidents [35,36].

#### 2.2.1. Bicycle Models

Currently used bicycle models included key components: frame, wheels, fork, pedals, pedal shaft, seats, and saddle. Every part of the bicycle was modeled as rigid with inertia moment and mass and joint setting, all of whose exact values were taken from previous work [1]. Table 1 shows the information about the bicycle models.

#### 2.2.2. Vehicle Models

The cars included in the considered accident cases are typical small passenger cars. The MB vehicle models were obtained by modifying the existing similar model validated in our previous studies [20,37,38]. Modifications mainly include car mass and front part geometry, to match the features of the cars involved in the accidents (Table 1). We used the stiffness parameters of the front components following the previous research [39,40].

#### 2.2.3. Multi-Body Cyclist Models

The subject-specific MB cyclist models were obtained by scaling the 50th percentage male and 5th percentage female pedestrian MB models (Table 2) according to anthropometric data of the analyzed cyclists (Table 1), and such scaling was performed with a GEBOD (Generator of Body Data) module in a MADYMO software package [41].

### 2.3. Models for Head/Brain-Neck Injury Evaluation

#### 2.3.1. Finite Element—Multi-Body Coupled Cyclist Model

**Figure 1:**
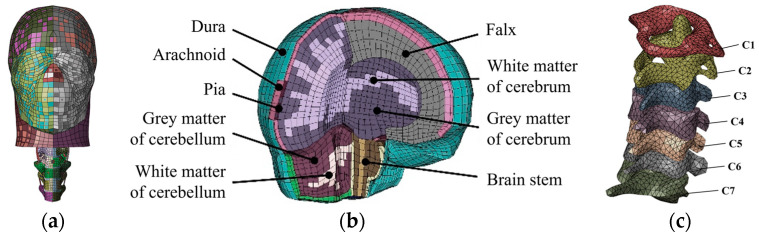
(**a**) THUMS head-neck FE model; (**b**) THUMS brain model; (**c**) THUMS neck model.

An MB-FE coupled pedestrian model was built in our group [33] to calculate the cyclist’s brain deformation and neck injury responses during the impact event. This model was constructed through the combination of the head-neck part (Figure 1a) taken from a THUMS (total human model for safety) FE human body model from the Toyota Central Research & Development Laboratories [42], and the other segments were taken from the adult pedestrian MB model. The THUMS brain model includes the cerebrum, cerebellum, brainstem, falx, tentorium, and meninges (Figure 1b). The THUMS neck model mainly includes the cervical spine (c1–c7), intervertebral disc, spinal canal, paravertebral muscles, and cervical vessels (Figure 1c). This combined MB-FE approach is motivated to leverage the MB model’s computation efficiency and the capabilities of the FE model to replicate the head-neck soft tissue responses in impact events [33].

#### 2.3.2. Helmet Models

The FE models of used helmets built in a previous study [43] were used, representing both high-price (helmets 1 and 3) and low-price (helmets 2 and 4) helmets in China. Each model included three key helmet components: an outer shell of ABS (acrylonitrile butadiene styrene) or PC (polycarbonate) plastic, a liner of EPS (expanded polystyrene) foam for energy-absorbing, and a chin strap. The keywords of “automatic surface to surface contact” were used for the definition of the contact between the head and the helmet. The key features of the used helmets and corresponding FE models are listed in Table 3.

#### 2.3.3. Final Simulation Model Set-Up

**Figure 2:**
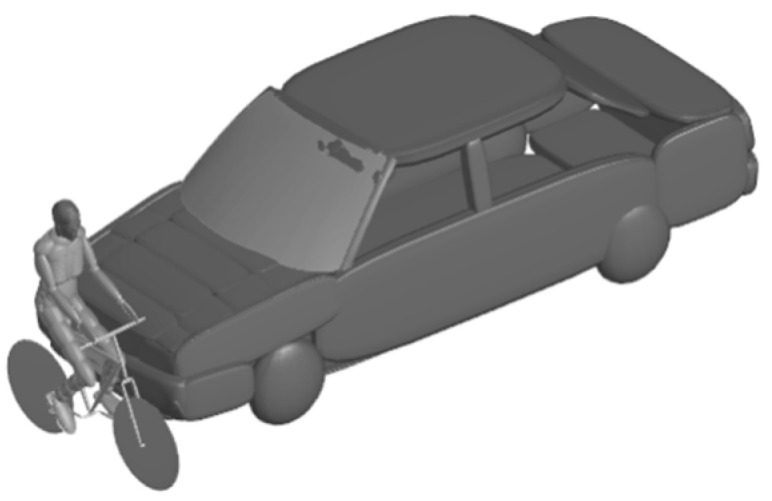
Set-up of car–cyclist impact case for the investigation of head/brain neck injury responses.

Selected accidents were initially reconstructed using the bicycle, vehicle, and full-scale MB cyclist models described above, and the initial boundary conditions of the accidents were output. The multi-body cyclists were then displaced with the MB-FE coupled models mentioned in Section 2.3.1, to obtain the head-brain and neck injury parameters. The simulation set-up is illustrated in Figure 2. All FE simulations in our study were performed using an LSDYNA R10.0 non-linear explicit dynamics code provided by LSTC (Livermore, CA, USA: http://www.lstc.com, accessed on 24 September 2023). The interface between the FE and MB models was achieved using the MB-FE coupling model from the MADYMO Version 7.5 software package.

### 2.4. Simulation Matrix

The five real-world car–cyclist impact cases were reconstructed for unhelmeted cyclists. Then, four different bicycle helmets were chosen to apply to the accidents. Taken together, 25 simulations were conducted in this study: 5 real-world car–cyclist impact cases involving unhelmeted cyclists + 5 cases × 4 helmets involving helmeted cyclists.

### 2.5. Injury Evaluation Criteria

#### 2.5.1. Head Injury Evaluation Criteria

The head injury criterion (HIC) is the most widely used head injury evaluation criterion in vehicle safety standards/programs [44]. It is related to both brain injury and skull fractures. The HIC is computed by the resultant linear acceleration of the head’s COG and duration [45]:

$$HIC={\left\{{\left(t_{2}-t_{1}\right)\left[\frac{1}{t_{2}-t_{1}}\int_{t_{1}}^{t_{2}}a(t)dt\right]}^{2.5}\right\}}_{max}$$

where *a*(*t*) represents the resultant linear acceleration of the head’s COG, and the time interval (*t*_2_ − *t*_1_) is chosen to maximize HIC over a maximum duration of 15 ms (referred to as HIC_15_).

The HIP is another head injury criterion based on the impact energy of the human head during a collision. The translational and rotational movements of the head’s COG are both considered by associating the kinetic energy change rate with the probability of head injury [46]. The calculation of the HIP is based on the following formula [45]:

$$HIP=ma_{x}\int a_{x}dt+ma_{y}\int a_{y}dt+ma_{z}\int a_{z}dt+I_{x}a_{x}\int \alpha_{x}dt+I_{y}a_{y}\int \alpha_{y}dt+I_{z}a_{z}\int \alpha_{z}dt$$

where *m* represents the head mass, set as 4.5 kg herein; *I_x_*, *I_y_*, and *I_z_* represent the moment of inertia, set as 0.016 kgm^2^, 0.024 kgm^2^, 0.022 kgm^2^ herein; *a_x_*, *a_y_*, and *a_z_* (m/s^2^) represent the resultant linear acceleration of the head COG; $\alpha_{x}$, $\alpha_{y}$, $\alpha_{z}$ (rad/s^2^) represent the head COG resultant angular acceleration [47]. The HIP was designed only for a brain injury; nevertheless, Marjoux et al. studied its prediction capability for SDH and skull fracture [47].

The maximum principal strain (MPS) was widely used for the assessment of the brain injury severity, for instance, a diffuse axonal injury (DAI) [48]. As the extreme MPS calculated at a single Gauss integration point in the brain tissue may lead to modelling artefacts, the 98th percentile MPS (MPS_0.98_) was used for the current analysis.

The cumulative strain damage measure (CSDM) measures the cumulative volume portion of the brain tissue experiencing strain over a predefined threshold. Following Takhounts et al. [49], we used the threshold of 0.25 (referred to as CSDM_0.25_).

#### 2.5.2. Neck Injury Evaluation Criteria

Boström et al. [50] proposed a neck injury criterion (NIC), which demonstrates the relationship between the injury to the spinal cord tissue and the pressure gradients. It considers the relative horizontal acceleration and velocity between the top (C1) and bottom (T1) of the cervical spine [51], and its calculation formula is as follows [52]:

$$NIC=0.2a_{rel}+{V_{rel}}^{2}$$

where *a_rel_* and *V_rel_* are the relative horizontal acceleration and velocity between the top (C1) and bottom (T1) of the cervical spine. The constant 0.2 denotes the approximate length of the neck in meters.

Another neck injury criterion, N_ij_, proposed by the National Highway Traffic Safety Administration (NHTSA), is also used to evaluate severe neck injuries [52]. N_ij_ is an index combination of axial force (*F_z_*) and bending moment (*M_y_*) measured at the cervical vertebrae. It is the most commonly used neck injury criterion, which can be expressed as the summary of the normalized loads and moment [53]:

$$N_{ij}=\frac{F_{z}}{F_{int}}+\frac{M_{y}}{M_{int}}$$

where *F_z_* represents the axial load, *F_int_* represents the critical intercept value of the load used for normalization, *M_y_* is the flexion/extension bending moment, and *M_int_* is the critical intercept value for the moment used for normalization. The critical values of the axial force and bending moment are: *F_int_* (tension) = *F_int_* (compression) = 4500 N; *M_int_* (flexion) = 310 Nm; and *M_int_* (extension) = 125 Nm [52].

## 3. Results

### 3.1. Cyclist Kinematics

**Figure 3:**
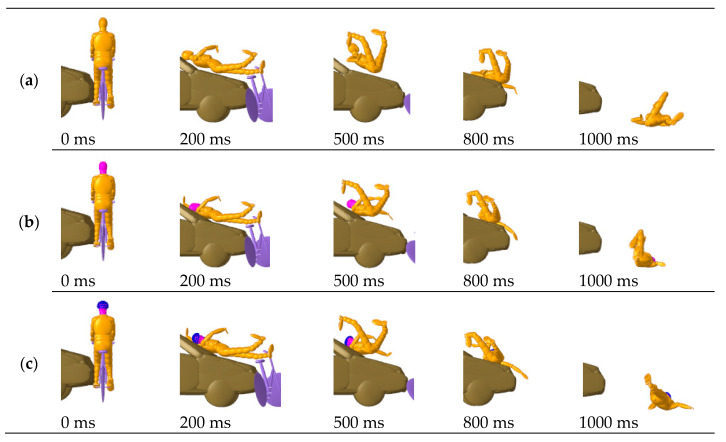
Predicted cyclist kinematics for Case 1: (**a**) MB accident reconstruction; (**b**) Simulation using the MB-FE coupled cyclist model; (**c**) Simulation using the MB-FE coupled cyclist model with helmet.

**Figure 4:**
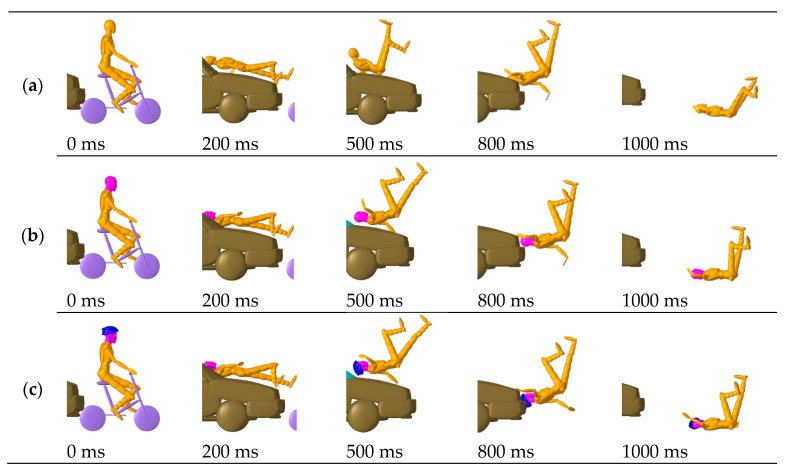
Predicted cyclist kinematics for Case 5: (**a**) MB accident reconstruction; (**b**) Simulation using the MB-FE coupled cyclist model; (**c**) Simulation using the MB-FE coupled cyclist model with helmet.

In the five analyzed cases, Case 1 and Case 5 correspond to two typical car-to-bicycle collision conditions: the vehicle directly impacts the side of the cyclist and then directly rear-ends them; Figure 3 and Figure 4 respectively show the dynamic impact configuration of the cyclist predicted in these two cases. It can be found that the cyclist’s head first collides with the front structure of the vehicle (windshield) and then collides with the ground twice at 1000 ms. At the same time, the overall kinematic responses of the three simulated cyclists in each case are consistent.

By comparing the results across different rows in Figure 3 and Figure 4, it can be noted that the impact configurations (1. full-scale MB accident numerical reconstruction; 2. simulation using MB-FE coupled cyclist models; 3. simulation with a cyclist wearing helmet 1) of the simulated accidents showed no significant difference in kinematics. The kinematic responses of the TNO model and the MB-FE coupled model are virtually comparable, and the addition of a helmet has little effect on the impact trajectories.

### 3.2. Head/Brain and Neck Injuries of the Cyclist

#### 3.2.1. Head Injury Criteria (HIC)

**Figure 5:**
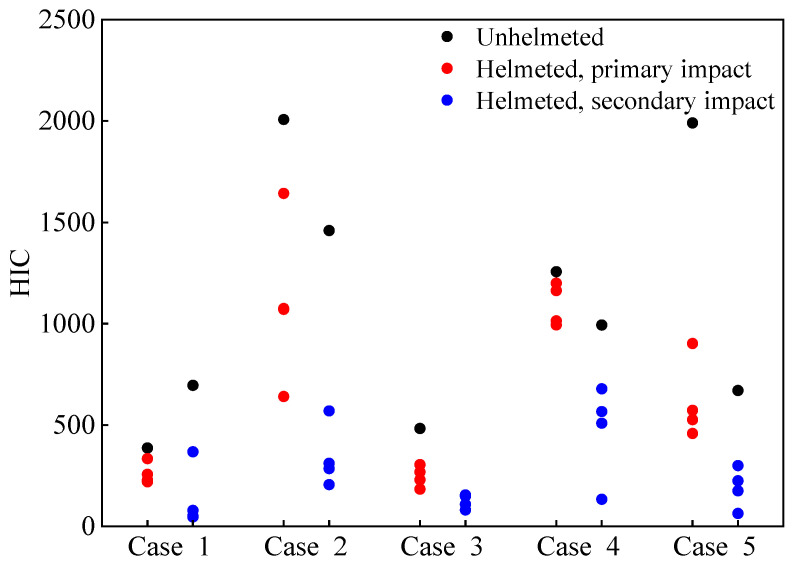
Predicted head injury criterion HIC_15_ for unhelmeted and helmeted cyclists in analyzed car–cyclist impact cases. Four different helmets are investigated.

The predicted head injury criterion (HIC_15_) at both the primary and secondary impact stages can be seen in Figure 5. The results show that, in general, the predicted HIC_15_ values at the first impact are higher than those at the secondary impact, with the only exception being Case 1. The introduction of a helmet substantially reduces the predicted HIC_15_, especially upon the secondary impact.

#### 3.2.2. Head Impact Power (HIP)

**Figure 6:**
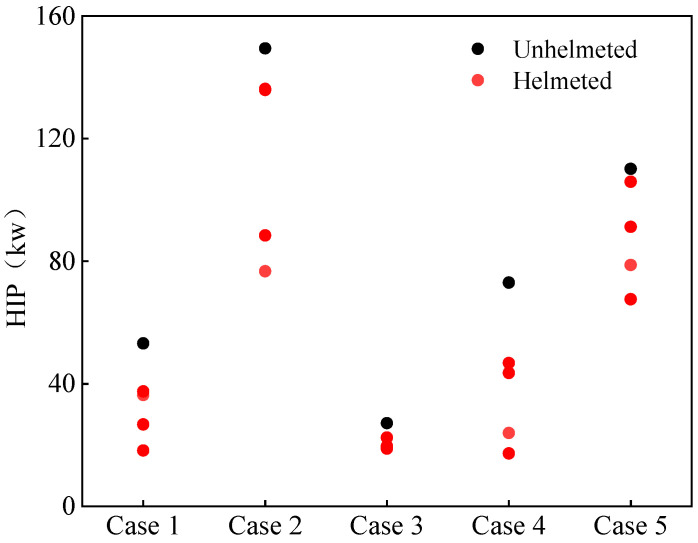
Predicted head impact power HIP for unhelmeted and helmeted cyclists in analyzed car–cyclist impact cases. Four different helmets are investigated.

The HIP considers both the translational and the rotational movement of the head. It was concluded in previous work [54,55] that wearing a helmet can increase the rotational acceleration. Figure 6 demonstrates the HIP index of all simulations covering all five selected accident cases and the inclusion of four different helmets. It can be observed that, for all the accident cases, the prediction values for helmeted cyclists are lower than for their unhelmeted counterparts.

#### 3.2.3. Cumulative Strain Damage Measure (CSDM)

**Figure 7:**
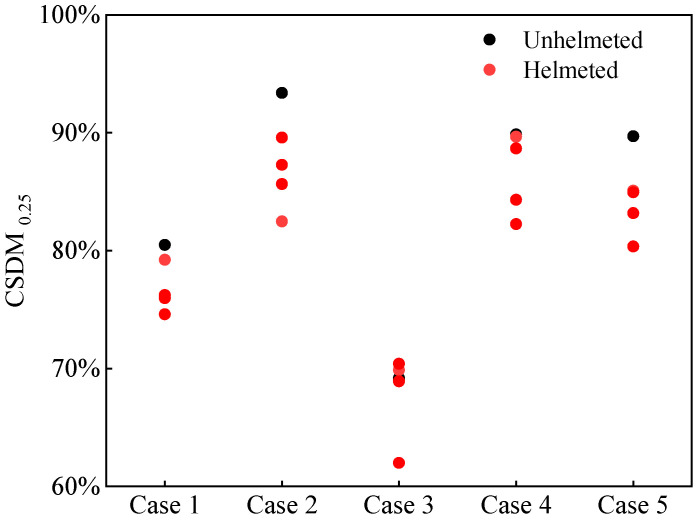
Predicted cumulative strain damage measure CSDM_0.25_ for unhelmeted and helmeted cyclists in analyzed car–cyclist impact cases. Four different helmets are investigated.

The CSDM was identified to correlate with the occurrence of brain injury. It measures the cumulative volume portion of the brain tissue experiencing a principal strain over a predefined threshold [49]. A level of 25% was chosen in this study, referred to as the CSDM_0.25_. As shown in Figure 7, wearing a helmet reduces the CSDM_0.25_ in almost all accidents (Figure 7) with the extent of reduction depending on the helmet types.

#### 3.2.4. Maximum Principal Strain (MPS)

**Figure 8:**
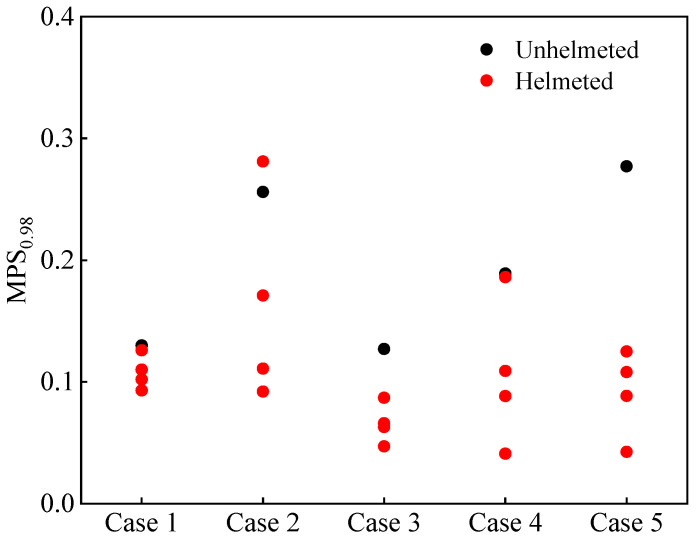
Prediction of the calculated 98th percentile maximum principal strain (MPS_0.98_) of brain tissue for unhelmeted and helmeted cyclists in analyzed car–cyclist impact cases. Four different helmets are investigated.

Figure 8 shows the computed MPS_0.98_ for all simulation cases. Similar to the analysis based on the criteria HIC and HIP, the results show that including a helmet lowers the predicted MPS_0.98_, and this reduction is more pronounced compared to the scenario where the CSDM_0.25_ is used for evaluation (see Figure 7).

#### 3.2.5. Neck Injury Criteria (NIC)

**Figure 9:**
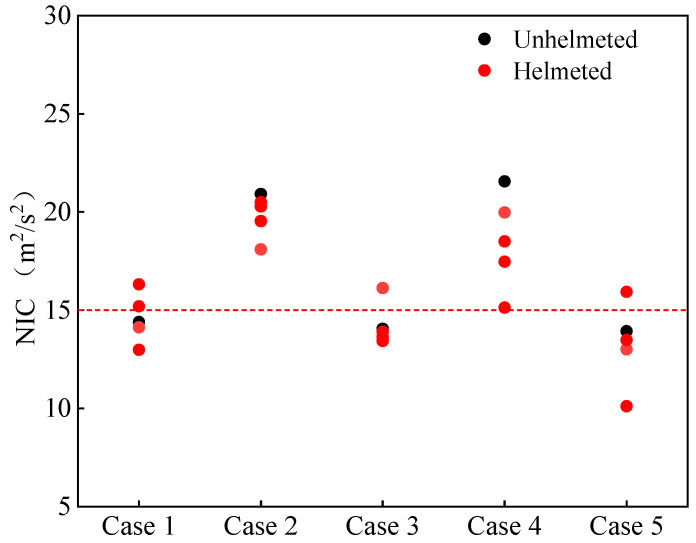
Neck injury criterion NIC value for unhelmeted and helmeted cyclists in car–cyclist collisions. The red line indicates the NIC threshold of 15 m^2^/s^2^ reported in the literature [56]. Four different helmets are investigated.

NIC is a neck injury index calculated based on the velocity and acceleration of the cervical vertebrae. The threshold value of the NIC is 15 m^2^/s^2^ [56]. As seen in Figure 9, for cases 2 and 4, the inclusion of helmets, regardless of the helmet type, lowers the calculated NIC values, although all remain beyond the threshold. For cases 1, 3 and 5, the reduction in NIC values depends on the selection of helmets, and the increase in the predicted NIC is observed for certain configurations.

#### 3.2.6. N_ij_

**Figure 10:**
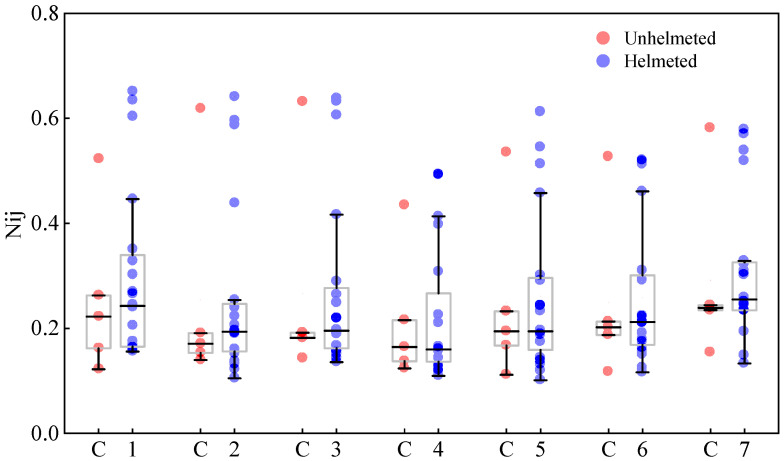
The N_ij_ value of all pieces of cervical spine for cyclists with and without a helmet in car–cyclist collisions.

Figure 10 shows the predicted N_ij_ values of seven cervical vertebrae C1–C7 (see Figure 1c) of cyclists in the five analyzed car–cyclist impact cases. In general, when the cyclist is not wearing a helmet, the distribution of the predicted N_ij_ is quite similar by accident case, with one case where it is remarkably higher than in other cases. It can also be observed that there is no significant difference in the predicted N_ij_ values between the unhelmeted and helmeted cyclists, and the use of helmets even leads to a slight rise in the predicted values. However, the predictions from all simulations are under the reported N_ij_ threshold of 1 [52].

#### 3.2.7. Neck Force and Bending Moment

**Figure 11:**
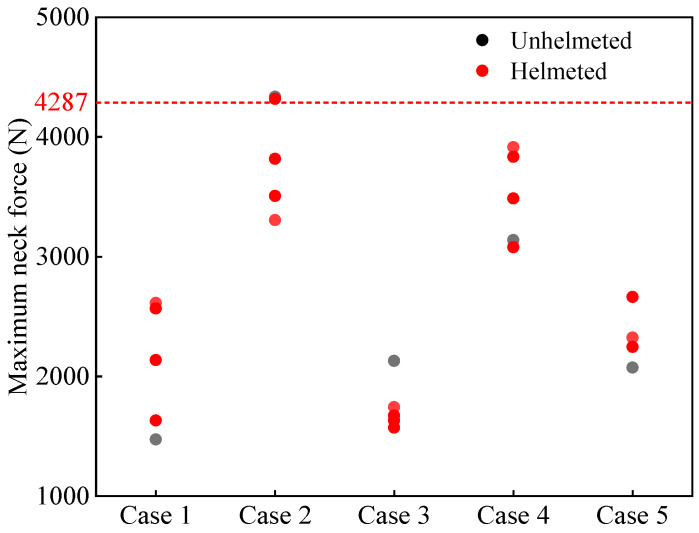
Maximum neck force for both unhelmeted and helmeted cyclists in car–cyclist impact cases. The red line indicates the neck force threshold of 4287 N reported in the literature [57]. Four different helmets are investigated.

**Figure 12:**
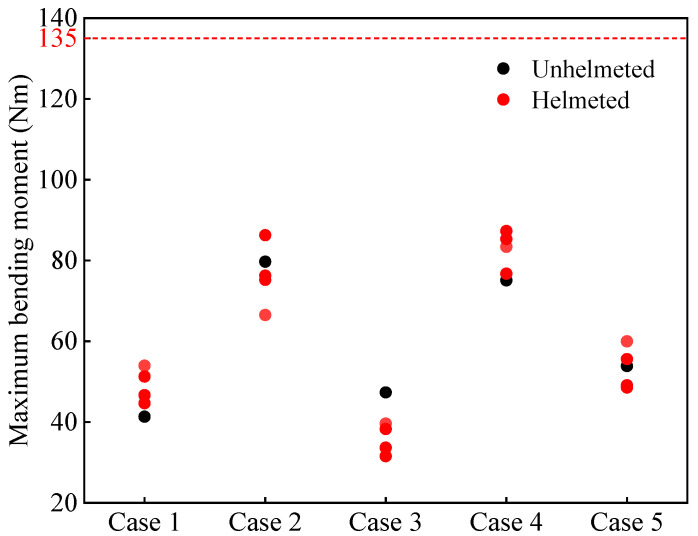
Maximum neck bending moment for both unhelmeted and helmeted cyclists in car–cyclist impact cases. The red line indicates the neck bending moment threshold of 135 Nm reported in the literature [57]. Four different helmets are investigated.

Figure 11 and Figure 12, respectively, present the maximum neck force and bending moment of all cyclists. According to the literature, the neck force threshold is 4287 N, and the neck bending moment threshold is 135 Nm [57]. As seen in Figure 11, the helmet tends to increase the neck force of the cyclists, although the predicted neck force does not exceed the reported threshold of 4287 N. As for the bending moment, the effect of the helmet depends on the helmet type. Overall, the increase/decrease in the predictions is not significant. Moreover, all predicted bending moments are lower than the reported threshold of 135 Nm.

### 3.3. Risk Assessment of the Cyclist’s Head/Brain Injury

In this study, we also investigated the head/brain injury risk to cyclists with and without a helmet in all analyzed accidents by using the head/brain injury risk curves described in the literature [47,48,49,58,59]. We divided the head/brain injury evaluation criteria into two groups: criteria based on head kinematics including HIC_15_ and HIP, and those based on brain tissue deformation including CSDM_0.25_ and MPS_0.98_.

#### 3.3.1. Injury Criteria Based on Head Kinematics

**Figure 13:**
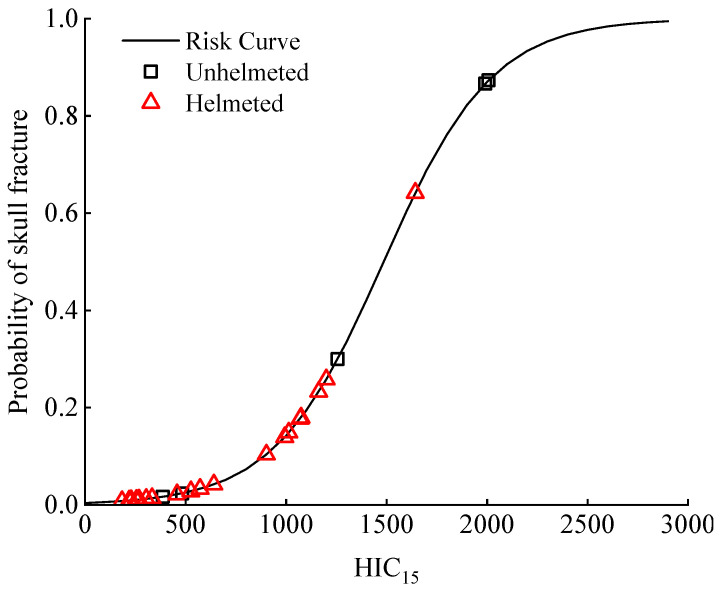
Distribution of the risk of skull fracture in unhelmeted and helmeted cyclists in all analyzed car–cyclist impact cases. The calculation is based on the skull fracture risk curve reported in the research by Yoganandan et al. [59]. Four different helmets are investigated.

In order to link the calculated HIC_15_ to the skull fracture risk to cyclists, we utilized the skull fracture risk curve developed by Yoganandan et al. [59]. The first impact was solely considered as the numerical results indicated that it produces a higher HIC_15_ compared to the ground impact (Figure 5). As seen in Figure 13, in the analyzed accidents, the unhelmeted cyclists had up to 85% probability of the occurrence of a skull fracture, and helmet use could lower such a risk.

**Figure 14:**
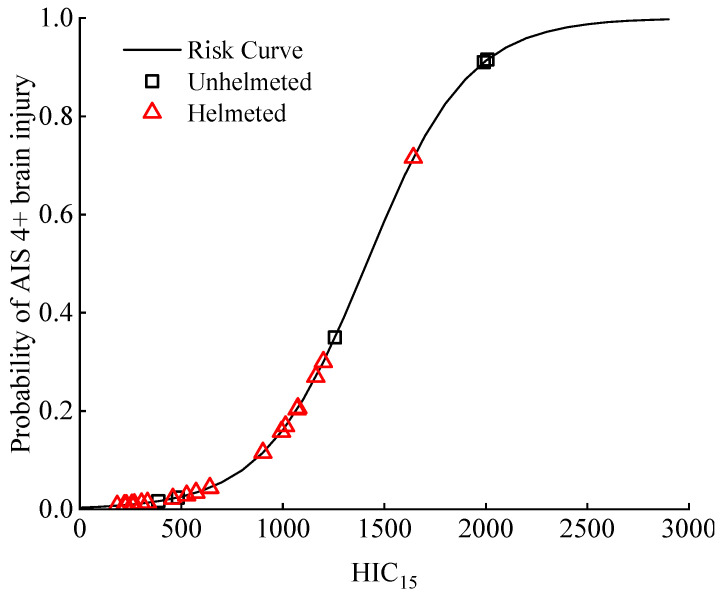
Distribution of the probability of severe (AIS 4+) brain injury to helmeted cyclists in the selected accident cases. Such a risk is computed from the HIC_15_ via the risk curve reported in the literature [59]. Four helmets are investigated.

The HIC_15_ was also reported to correlate with the human body brain injury risk, as demonstrated by the risk curve reported in the literature [59], that relates the HIC_15_ with the probability of experiencing a given trauma level indexed by the AIS (abbreviated injury scale: a common injury index to quantify the severity of a human body injury [58]; we concentrated on the severe brain injury categorized as AIS 4+ [58]). The probability distribution of an AIS 4+ brain injury was similar to that of a skull fracture (Figure 13 and Figure 14), which means a helmet could significantly lower the risk of a severe brain injury (Figure 14).

**Figure 15:**
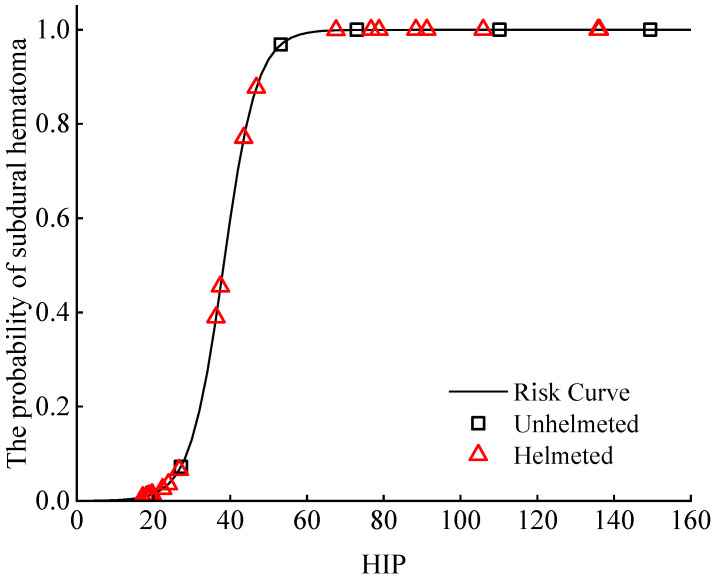
Distribution of the risk of subdural hematoma to cyclists with or without a helmet in car–cyclist collisions. The risk of subdural hematoma was determined from the HIP using the injury risk curve by Marjoux et al. [47]. Four different helmets are investigated.

The HIP is usually used to assess the risk of subdural hematoma (SDH) [47]. Figure 15 shows the distribution of the probabilities of SDH in cyclists calculated from the risk curve developed by Marjoux et al. [47]. For the cases of extremely high risk of SDH, the risk is still significant in spite of the reduction in the predicted HIP estimated from helmeted impacts. For the less severe cases (but not the one with a lower than 10% risk), a clear decrease in such a risk can be observed via the inclusion of a helmet.

#### 3.3.2. Injury Criteria Based on Brain Tissue Deformation

**Figure 16:**
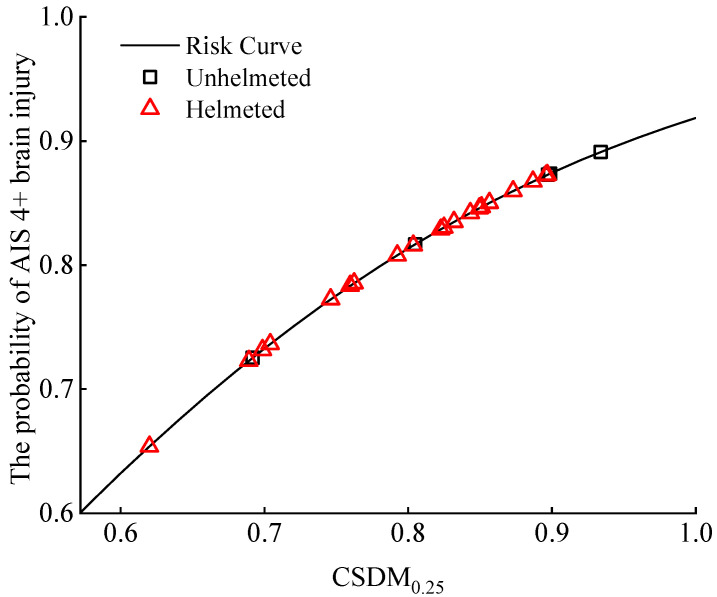
Distribution of the risk of brain injury with AIS 4+ severity in cyclists with or without a helmet in car–cyclist collisions. The risk of AIS 4+ brain injury was determined from CSDM_0.25_ using the injury risk curve by Takhounts et al. [48]. Four different helmets are investigated.

Takhounts et al. [48] developed an injury risk curve to link the CSDM_0.25_ with the probability of an AIS 4+ brain injury, as shown in Figure 16. Although the introduction of the helmet brings a noticeable decrease in the distribution of the risk of an AIS 4+ brain injury related to the CSDM_0.25_ for most of the analyzed accident cases, the risks of an AIS 4+ brain injury to the cyclists involved in the studied accident cases are still quite high (not less than 60%) irrespective of helmet use.

**Figure 17:**
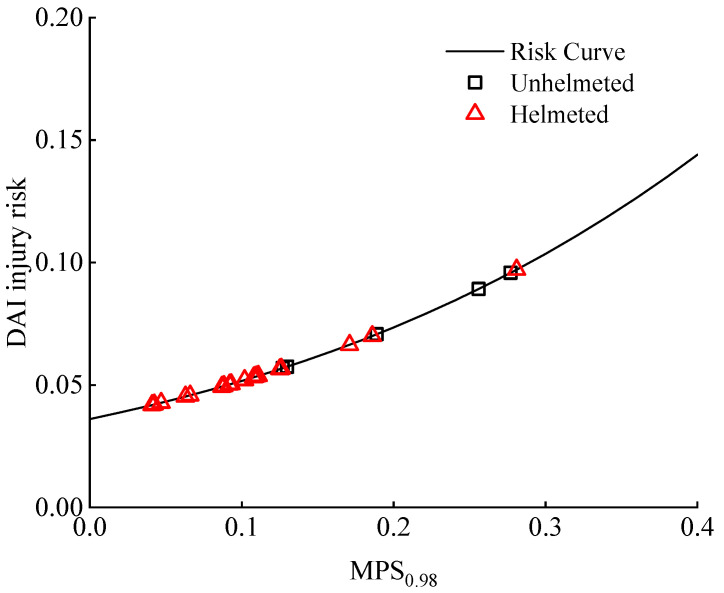
Distribution of DAI risk to both unhelmeted and helmeted cyclists in the analyzed accident cases. Such a risk was calculated from the MPS_0.98_ via the risk curve reported in the literature [49]. Four helmets are investigated.

Similar to the above analysis, we conducted an evaluation of a DAI risk related to the distribution of the MPS_0.98_ of the cyclists. The risk curve reported in the literature [49] relates the MPS with the risk of a DAI (see Figure 17). Clearly, the DAI risks calculated from the MPS_0.98_ for helmeted cyclists were generally lower than those of the unhelmeted cyclists. However, such a reduction was much less significant when the risks of an AIS 4+ brain injury were evaluated with a HIC (Figure 14), as the initial DAI risk to the unhelmeted cyclists in the cases was already very low (Figure 17).

## 4. Discussion

### 4.1. Head/Brain-Neck Injury

From the analysis of the predicted HIC_15_ and the related risk of a skull fracture and AIS 4+ brain injury (Figure 5, Figure 13 and Figure 14), it was indicated that a helmet can effectively reduce the predicted HIC_15_ values and consequently the skull fracture risk to the cyclist as well as the AIS 4+ brain injury (determined from the risk curves using the HIC_15_) in car–cyclist impact cases. The results obtained in our study confirm the commonly accepted view in previous papers [14,15,60,61,62].

The HIP relies on two influencing factors: linear acceleration and rotational acceleration. Therefore, compared with the HIC solely based on linear acceleration, the HIP provides an additional insight into head injuries to cyclists. From the results of the HIP analysis (Figure 6), it can be seen that helmets can significantly reduce the HIP predictions. However, such a reduction is not as large as that for the HIC_15_, which may be due to the increase in the rotational acceleration of the cyclist’s head. A similar observation was also noted in previous studies [43,55]. Assuming the HIP to be an effective predictor of SDH, our results showed that wearing a helmet decreases the risk of SDH to a varying extent based on the five analyzed accident cases.

The CSDM_0.25_ predicted using the THUMS brain FE model [63] is also currently used for the indicator of the risk of cyclist brain injury. From the results of the CSDM_0.25_ and the corresponding risk of an AIS 4+ brain injury based on the existing injury risk curve (Figure 7 and Figure 16), it can be observed that both the predicted CSDM_0.25_ and the calculated severe brain injury risk are extremely high in typical car–cyclist impact cases, with the probability of an AIS 4+ brain injury over 60% for both unhelmeted and helmeted cyclists.

Our results also showed that wearing a helmet can considerably lower the MPS_0.98_ level and the MPS-related DAI risk in nearly all analyzed car–cyclist impact cases (Figure 7 and Figure 8). Several existing studies used the deformation of brain tissue to assess the safety performance of helmets against head injury. For instance, using the FE human body head model, Fahlstedt et al. [64] found that helmets could bring down the calculated MPS level by up to 43%, which matches well with our results.

### 4.2. Neck Injury

The cervical spine contains seven segments from C1 to C7 cervical vertebrae. Li et al. [52] conducted a literature review research in the field of human body neck injury biomechanics and protection in vehicle crashes, and found that for all impact configurations, the C4 to C7 segments were at the highest risk of ligament and disc injury, i.e., the lower parts of the cervical spine. However, the analysis results from our research indicated that C1 and C7 had a greater risk of neck injury, and C4 exhibited the lowest risk of injury. This may be because C1 is located at the top of the cervical spine and is directly subjected to the force and speed transmitted from the head, while C7 is at the bottom of the cervical spine, which needs to undergo the entire weight of the head-neck complex. Moreover, C7 is the farthest segment away from the COG of the head; thus, it receives a large bending moment.

The neck injury mechanism is highly complicated. In traffic accident reports, most cases lack any external evidence of a neck injury, so it is easy to ignore a serious neck soft tissue injury [27]. Previous research found that helmet use does not affect the neck injury [10,27,61,65], but these were conclusions drawn from the statistical results of accident reports that did not record the neck soft tissue injury. In this paper, the relevant neck injury was investigated through FE simulation analysis, and it was found that wearing a helmet possibly increases the neck force and bending moment in some cases, although not too significantly (Figure 11 and Figure 12).

### 4.3. Effect of Helmet Price

**Figure 18:**
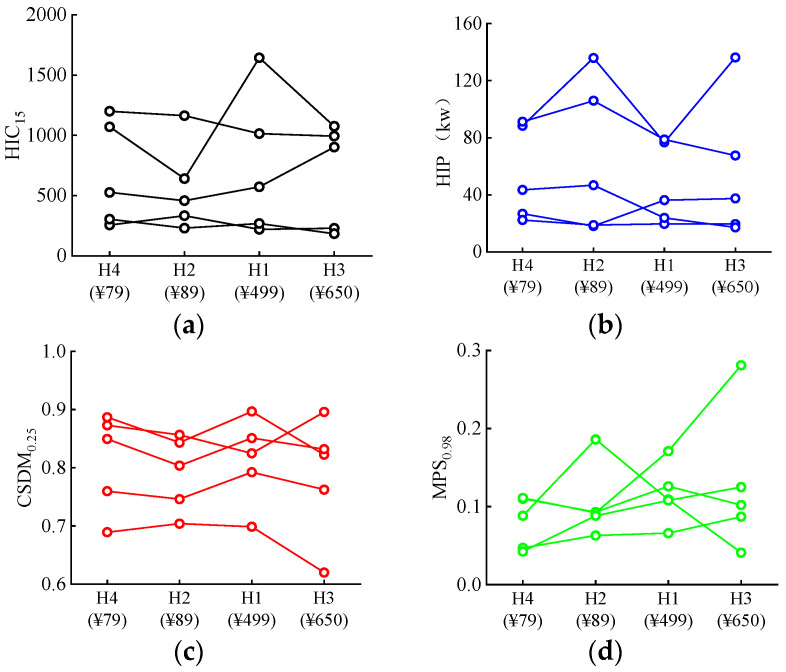
Effect of helmet price on the head/brain injury to cyclists: (**a**) HIC_15_; (**b**) HIP; (**c**) CSDM_0.25_; (d) MPS_0.98_.

The helmets analyzed in the current study varied in price (see Table 3). No evident correlation was found between the predicted head/brain injury criteria and the helmet price (Figure 18). Even relatively low-cost helmets (helmets 2 and 4) performed slightly better than the more expensive ones in reducing the predicted head injury criteria, such as some cases for HIC_15_, CSDM_0.25_ and MPS_0.98_ (Figure 18a,c,d). This is in line with the findings of our previous work [43].

**Figure 19:**
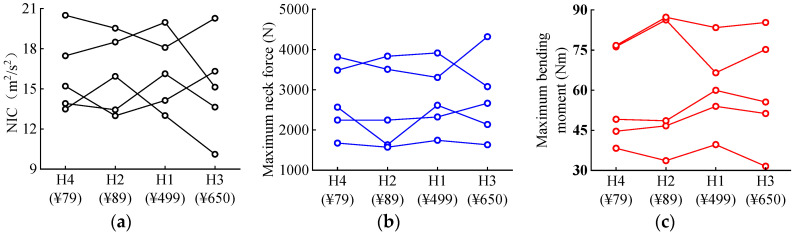
Effect of helmet price on the neck injury to cyclists: (**a**) NIC; (**b**) Maximum neck force; (**c**) Maximum bending.

Furthermore, there was no significant association between neck-related injury parameters and the helmet price (Figure 19). In some cases, cheaper helmets (H2 and H4) achieved more reduced neck injury predictions than expensive helmets (H1 and H3).

### 4.4. Study Limitations

Two major limitations of this study need to be highlighted. First, we only analyzed four helmets and five car–cyclist impact cases. Although this helps us get a better understanding of the helmet efficacy in protecting the head/brain and neck, a more in-depth analysis involving more helmet types and impact cases is needed, further reinforcing the generality and robustness of the conclusion of the current study. Second, we did not consider the variation in friction between different helmets and the scalp, as the same friction of coefficient in the helmet–scalp interface was used. How the friction between the helmet and the scalp and the stretching force of the chin strap affect the helmet’s efficacy is another possible direction for future work.

## 5. Conclusions

We investigated the performance of four off-the-shelf bicycle helmets in preventing the head/brain and neck injury to cyclists by computationally reconstructing five real-world car–cyclist impact events. From the limited number of cases we analyzed, the analysis results lead to the following conclusions:

- Overall, bicycle helmets can provide substantial protection to cyclists in the analyzed accident cases. Specifically, helmets can remarkably lower the risk of both severe (AIS 4+) brain injury and skull fracture, as assessed by the predicted HIC, while a relatively limited decrease in AIS 4+ brain injury risk can be achieved in terms of the analysis of the CSDM_0.25_. Wearing a helmet also lowers the risk of subdural hematoma (by HIP) and diffuse axonal injury (by MPS_0.98_) in the cyclist, while such an effect is less pronounced than that for an AIS 4+ brain injury and skull fracture.
- The protective ability of a helmet against a neck injury differs according to the accident scenario and helmet type. The C1 and C7 of the cyclist’s cervical spine are more likely to be severely injured, while the C4 is generally less affected. Based on the analyzed neck injury criteria and the limited accident cases, the inclusion of a helmet seems to provide some level of protection to the neck of the cyclists in some cases and induces a slight increase in the predicted criteria in other cases.

## Figures and Tables

**Table 1:** Specific information about five car–cyclist impact accidents analyzed in the current study.

Information		Case 1	Case 2	Case 3	Case 4	Case 5
Accident	Vehicle impact velocity (km/h)	28	32	26	35	40
	Bicycle moving velocity (m/s)	4.4	1.5	2.5	1.2	1.1
	Injury	Minor injury	Serious injury	Minor injury	Death	Serious injury
	Key image	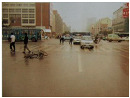	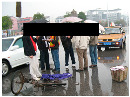	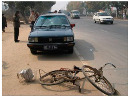	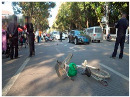	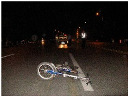
Bicycle	Wheel base (mm)	1130	950	1080	950	820
	Mass (kg)	19.5	15	19.5	20	10.5
	Tire radius (mm)	330	300	325	310	200
	Seat height (mm)	850	850	864	830	760
Vehicle	Car manufacturer	Geely	FAW-Volkswagen	Shanghai-Volkswagen	FAW-Volkswagen	Shanghai-Volkswagen
	Mass (kg)	1270	1105	1090	1565	1090
	Size (mm)	4150 × 1620 × 1450	4428 × 1660 × 1420	4546 × 1710 × 1427	4818 × 1843 × 1432	4546 × 1710 × 1427
Cyclist	Gender	male	male	male	male	female
	Height (cm)	168	165	170	165	161
	Weight (kg)	67	55	80	60	45
	Age	65	62	58	65	23

**Table 2:** Anthropometric information about the 50th percentage male and 5th percentage female TNO models used in this study.

	5th Percentile Female		50th Percentile Male	
Height (m)	1.53	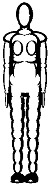	1.74	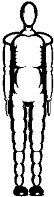
Weight (kg)	49.8		75.7	

**Table 3:** Information about the helmet models included in this study.

	Helmet	FE Model	Price (CNY)	Mass (g)	Element Number of the Helmet FE Model		Number of Nodes in the FE Helmet Model
					Triangular Elements	Tetrahedral Elements	
Helmet 1	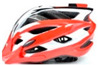	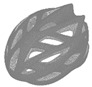	499	230	17,486	47,850	14,949
Helmet 2	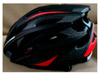	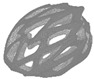	89	275	16,350	42,020	12,079
Helmet 3	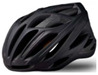	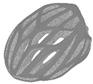	650	335	27,620	69,822	17,059
Helmet 4	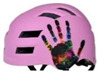	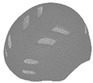	79	390	16,652	45,541	13,276

## Data Availability

Data sharing not applicable.
